# Antibacterial and Antibiofilm Activity of Different Species of *Fabiana* sp. Extract Obtained via Maceration and Ultrasound-Assisted Extraction against *Staphylococcus epidermidis*

**DOI:** 10.3390/plants12091830

**Published:** 2023-04-29

**Authors:** José Martínez Chamás, María Inés Isla, Iris Catiana Zampini

**Affiliations:** Laboratorio de Investigación de Productos Naturales (LIPRON), Instituto de Bioprospección y Fisiología Vegetal (INBIOFIV-CONICET-UNT), Facultad de Ciencias Naturales e IML, Universidad Nacional de Tucumán, San Lorenzo 1469, San Miguel de Tucumán PC:4000, Tucumán, Argentina; jmartinezchamas@gmail.com (J.M.C.); misla@csnat.unt.edu.ar (M.I.I.)

**Keywords:** *Fabiana* species, *Fabiana densa*, *Staphylococcus epidermidis*, ultrasound-assisted extraction antibacterial, antibiofilm

## Abstract

*Staphylococcus epidermidis* is an opportunistic pathogen that, under certain conditions, can induce aggravated infectious processes, mainly in immunosuppressed patients. Moreover, *S. epidermidis* is one of the leading causes of medical device- and implant-associated infections and is also recognized as a canonical biofilm producer. *Fabiana punensis*, *F. densa* and *F. patagonica* are three medicinal plants that grow in arid environments in Argentina (Altoandina, Puna, Prepuna and Monte regions). In this work, we studied the antimicrobial activity of alcoholic extracts of these plant species obtained via maceration (M) and ultrasound-assisted extraction (UAE) against *S. epidermidis*. In addition, the antibiofilm activity of the *F. densa* extract was also evaluated. It was found that the extracts obtained via M did not present differences with those obtained via UAE regarding the chemical profile. *F. densa* showed the lowest minimum inhibitory concentration (MIC) value (75 µg GAE/mL). At concentrations higher than the MIC, the extract induced the release of cellular constituents. At the concentration of 1/8× MIC, the extract inhibited biofilm formation by 78%, reducing metabolic activity by 67%. On the other hand, it presented a low percentage of preformed biofilm removal. In all assays, gallic acid (GA) has been used as a reference antimicrobial compound. Finally, it was shown via microscopy visualization that the extract reduces adhesion to hydrophobic and hydrophilic surfaces. Thus, *F. densa* extracts could potentially be used for the antibiotic treatment of infections produced by *S. epidermidis* or as an inhibitor agent of production biofilm, avoiding infections caused by medical devices.

## 1. Introduction

*Staphylococcus epidermidis* is a commensal Gram-positive coagulase-negative bacterium universally present in humans [1]. It is a bacterium capable of colonizing various areas of the human body such as skin, nostrils, head, and mucous membrane. Under certain conditions, *S. epidermidis* behaves as an opportunistic pathogen, worsening conditions in immunosuppressed patients such as patients with implanted medical equipment, chronically hospitalized, drug misusers, and patients with acquired immunodeficiency syndrome [2]. It is one of the pathogens that are commonly found in infections produced in premature infants during late-onset sepsis [3] and can complicate inflammatory processes in neonates such as bronchopulmonary dysplasia, white matter injury, and retinopathy of prematurity [4]. The application of invasive procedures in modern medicine has significantly favored the development of infections caused by nosocomial pathogens, mainly in the setting of prosthetic devices such as intravenous catheters, prosthetic joints, and heart valves [5,6]. Infections caused by *S. epidermidis* are due in many situations to its ability to form a biofilm on biotic surfaces (host tissue) or inert surfaces (medical implants, catheters, prostheses) [7,8]. Biofilm is a virulence factor that allows planktonic bacteria to adhere to surfaces and generate cellular aggregates, thus forming sessile microcolonies surrounded by a polymeric extracellular matrix formed mainly by proteins, carbohydrates, lipids, and DNA, enhancing the host’s immune system’s reaction as well as its resistance to antibiotics [9]. The worldwide distribution of three lineages of *S. epidermidis* of the multi-drug-resistant (MDR) clonal complex 2 and their adaptation to hospitals has been reported, which has presented a broad spectrum of resistance to antibiotics such as β-lactams, rifampicin, and vancomycin [10]. The presence of these pathogens, both sensitive and resistant, raises the clinical importance and the need to seek medical alternatives to treat this type of infection.

Since ancient times, plants have been utilized to treat illnesses [11]. They represent a renewable and economic source of antimicrobials. The presence of metabolites, which are mostly formed during the secondary metabolism of plants, is what gives plant-derived extracts their antibacterial properties. Although not necessary for development, these metabolites are crucial for defense, protection, and competitiveness, especially phenolic derivatives [12,13]. Infections can be combated using antibiotics, which, in turn, prevent cell wall formation, promoting membrane instability, and blocking DNA replication or protein synthesis [14]. Another alternative approach is anti-virulence therapy, which consists of combating virulence factors such as biofilm, quorum sensing, motility, toxins, etc. This therapy leads to a less severe infection and allows the host to have an immune response. The advantage of anti-virulence therapy is that it affects factors that are not necessary for survival or growth; therefore, it induces less selective pressure, which makes resistance development difficult [15], and it also has more target molecules and thus other mechanisms of action [16].

The *Fabiana* genus of South America belongs to the Solanaceae family. It grows in dry areas of the mountainous regions of the southern Andes. In Argentina, some species located in the Altoandina, Puna, Prepuna and Monte regions in northwestern Argentina have been described. Among them, one can find *F. bryoides*, *F. densa, F. patagonica* and *F. punensis*. All of them are used traditionally in medicine as anti-inflammatory and antiseptic, as well as as materials for construction, fodder, and spiritual activities [17,18,19,20,21]. Antimicrobial activities of alcoholic extracts of different *Fabiana* species against Gram-positive and Gram-negative pathogens have been reported, as well as diuretic, antioxidant, and anti-inflammatory activity [22,23,24,25,26].

The extraction method is a key step for the separation of molecules of interest within a complex matrix. Some of the conventional methods such as maceration (M) or Soxhlet have some disadvantages such as longer extraction time, extra use of solvents or higher energy consumption. Ultrasound-assisted extraction (UAE) is an unconventional extraction method that has some advantages, such as less time and energy consumed, less solvent used, and improved extraction yields, and is considered a clean and environmentally friendly technology [27,28].

The aim of this work was to obtain alcoholic extracts from different species of *Fabiana* collected from the northwestern region of Argentina using conventional (maceration) and non-conventional (UAE) extraction methods, to select the one with the best antimicrobial activity against *S. epidermidis,* and to evaluate its ability to inhibit the biofilm formation.

## 2. Results

### 2.1. Comparison of Ultrasound-Assisted Extraction (UAE) and Maceration

From the extracts obtained via M, the *Fabiana patagonica* extract showed the highest content of total phenolics compounds (TPC), while from the extracts obtained via UAE, *F. patagonica* and *F. punensis* extracts showed higher content of TPC than *F. densa* (Table 1). Both extraction methods only showed significative differences in the content of extracted TPC from *F. patagonica*. However, this content was only 18% higher than that obtained via UAE. On the other hand, the content of total flavonoids (TF) was significantly higher in *F. punensis*, *F. densa* and *F. patagonica* extracts obtained via M compared to those obtained via UAE, 23, 35 and 48%, respectively. It was also observed that, as with the TF content, the dry extract yield was significantly higher for the extracts obtained via M compared to those obtained via UAE, 18% (*F. punensis*), 20% (*F. densa*) and 55% (*F. patagonica*).

**Figure 1:**
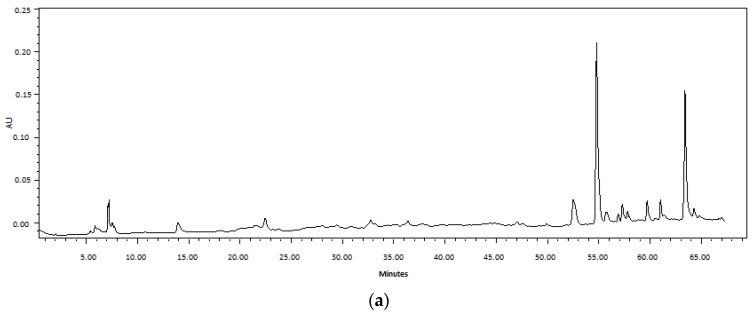

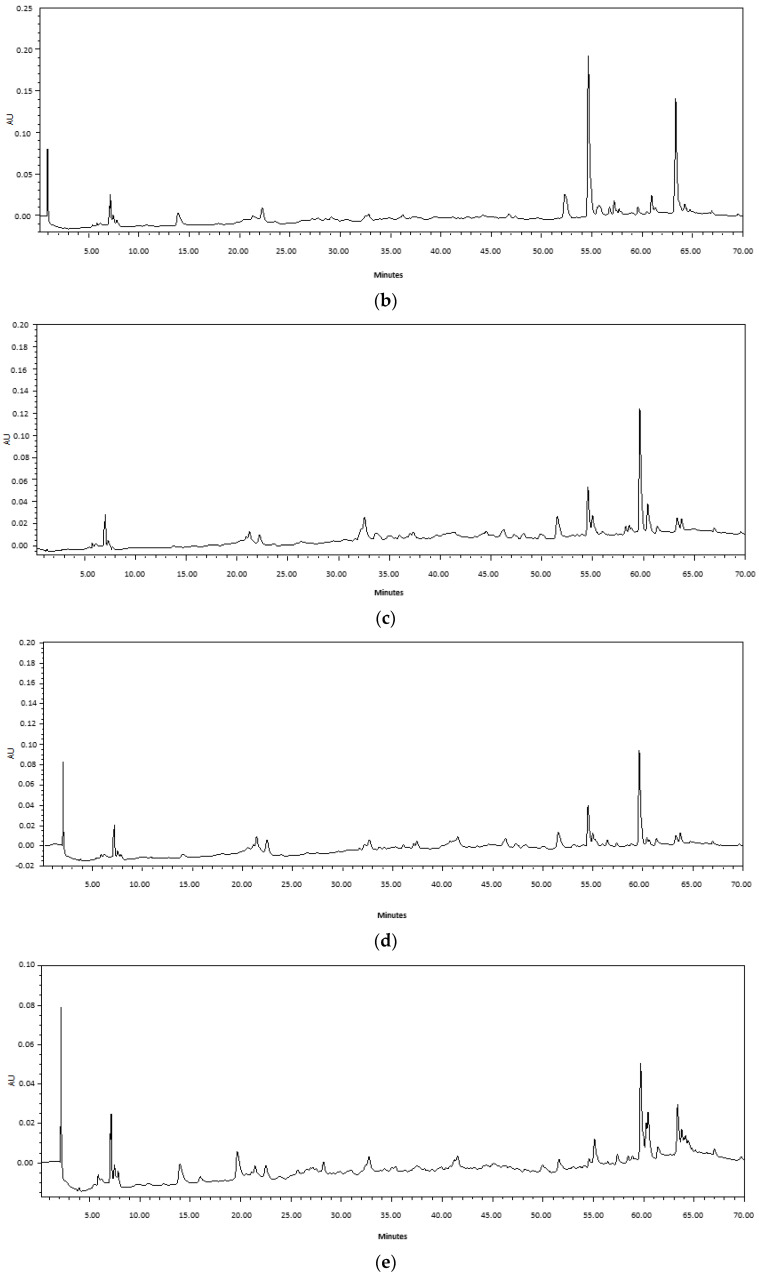

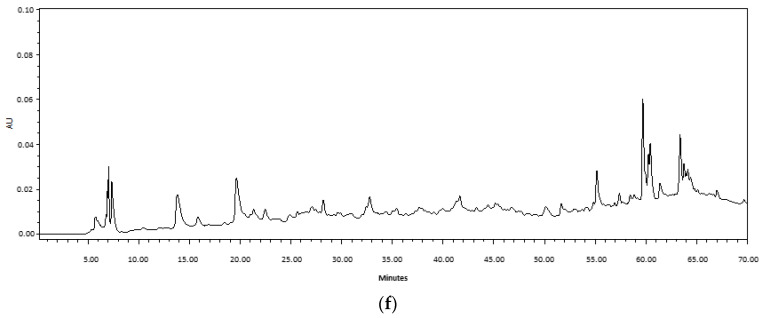
HPLC profile of Fabiana extracts obtained via maceration and UAE. (**a**) *F. punensis*—maceration; (**b**) *F. punensis*—UAE; (**c**) *F. densa*—maceration; (**d**) *F. densa*—UAE; (**e**) *F. patagonica*—maceration; (**f**) *F. patagonica*—UAE. The fingerprints were registered at 254 nm.

On the other hand, different HPLC profiles were found for each plant species; however, no differences were found in the HPLC profiles between extracts obtained via M and UAE for each plant species. In all cases, a high content of less polar compounds was observed (Figure 1).

### 2.2. Antimicrobial Activity

#### 2.2.1. Profile of Antibiotic Resistance of *S. epidermidis*

According to the zone diameter breakpoints provided by the Clinical and Laboratory Standards Institute guidelines M100- Ed. 32 [29], the *S. epidermidis* INBIOFIV S11 strain showed resistance to penicillin, ampicillin, erythromycin, and gentamicin, and was sensitive to tetracycline, chloramphenicol, rifampicin, and oxacillin. No inducible resistance to clindamycin was observed.

#### 2.2.2. Effect of *Fabiana* Species on Growth of *S. epidermidis*: Reduction in Cell Viability and Minimum Inhibitory Concentration (MIC)

Antimicrobial activity against *S. epidermidis* was demonstrated by *Fabiana* species. There were no differences observed in the MICs of the extracts obtained via M and UAE using the macrodilution method. The *F. densa* extract displayed the lowest MIC values (75 µg GAE/mL), followed by the *F. patagonica* extract with double the MIC values of *F. densa* (150 µg GAE/mL). Finally, *F. punensis* exhibited the highest MIC values (1200 µg GAE/mL). As *F. densa* exhibited the highest antimicrobial potency, the decision was made to continue the studies using *F. densa* extracts.

**Figure 2:**
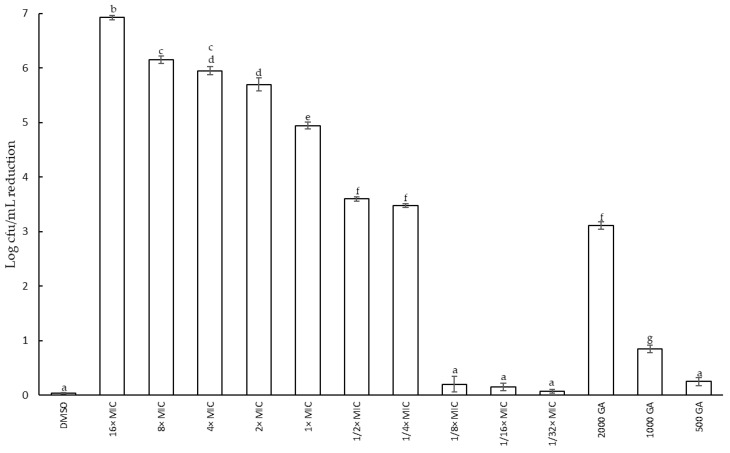
Reduction in cell concentration of *S. epidermidis* at different concentrations of the extract of *F. densa* obtained via UAE and different concentrations of gallic acid (GA) (positive control) at 8 h of incubation. Different letters indicate significant differences (*p* < 0.05).

Figure 2 shows the reduction in cell viability at different concentrations of the *F. densa* extract obtained via UAE during 8 h of incubation at 37 °C. After 8 h of incubation, no significant changes were observed. Values above the MIC reduce the cell viability until 8 h of incubation have elapsed. A concentration of 16× MIC showed the higher reduction in cell concentration of 73.24% (log 6.86 CFU/mL). Extract concentration of 8× MIC and 4× MIC did not show significant differences at the same time and they both reduce it by 65.81 and 63.14%, respectively. No significant differences between 2× MIC and 4× MIC were evident at 8 h of incubation. On the other hand, sub-MIC concentration (1/2× MIC and 1/4× MIC) showed a similar effect to that of the concentration of 2000 µg/mL of GA, used as a reference compound. Although these three treatments did not show a decrease in cell concentration, they did show a slowdown in cell growth with respect to the control. Dilutions below 1/8× MIC and 500 µg/mL GA did not show differences with respect to the control, suggesting that, under these conditions, cell viability did not show inhibition. Additionally, DMSO (solvent) did not present any negative effect on cell viability. Cell viability also was determined in the presence of *F. densa* extract obtained via maceration. No differences were found in the growth curve assayed at the same concentration.

#### 2.2.3. Cell Constituents’ Release of *S. epidermidis*

**Figure 3:**
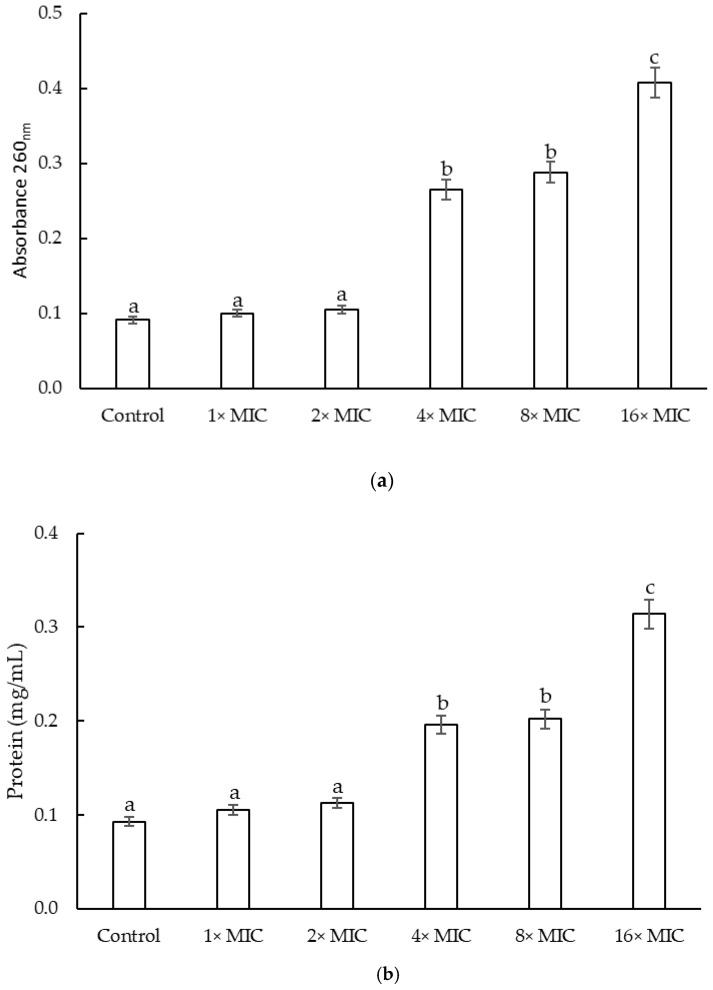
Release of cell material that absorbs at 260 nm at 4 h of incubation (**a**) and protein at 8 h of incubation (**b**) from *S. epidermidis* treated with *F. densa* extract. Means with different superscripts show significant differences for different *F. densa* extract treatments (*p* < 0.05).

The release of cellular constituents was determined via absorbance measurements at 260 nm (Figure 3a). The highest absorbance was detected at 4 h of incubation for extract concentrations of 16× MIC. The concentrations of 8× MIC and 4× MIC did not show differences at 4 h of incubation. For the 16× MIC and 8× MIC concentrations, a slight decrease in absorbance was observed at 24 h, while the absorbance at 4× MIC concentration remained constant until 24 h of incubation (data not shown). The concentrations of 2× MIC and the 1× MIC did not show differences with respect to the control. On the other hand, Figure 3b showed the protein content. A rapid increase in the content of released proteins was observed with the two highest concentrations assessed until 8 h of incubation (0.31 ± 0.0011 and 0.20 ± 0.0013 mg protein/mL for 16× MIC and 8× MIC, respectively). Then, the protein content remained constant for all concentrations. At the 4× MIC concentration, the release of the protein content was slower; only after 8 h of incubation did the protein content reach a protein content as that of the 8× MIC concentration. The concentrations of 2× MIC and MIC did not present significant differences between them for any of the times, and the concentration of protein released was significantly lower than the content of protein released by the other concentrations assayed (0.10 ± 0.0009 mg/mL).

### 2.3. Antibiofilm Activity of F. densa Extract

#### 2.3.1. Determination of the Minimum Inhibitory Concentration of *F. densa* Extracts on Biofilm Formation and on Metabolic Activity

According to the results observed in the viability curves obtained by counting colony on MH agar medium presented in the previous section, the *F. densa* extract concentrations between 1/8× MIC and 1/32× MIC and gallic acid concentration of 1000 and 500 µg/mL were chosen to evaluate the effect on biofilm formation because, at these sub-MIC concentrations, *S. epidermidis* growth was unaffected.

**Figure 4:**
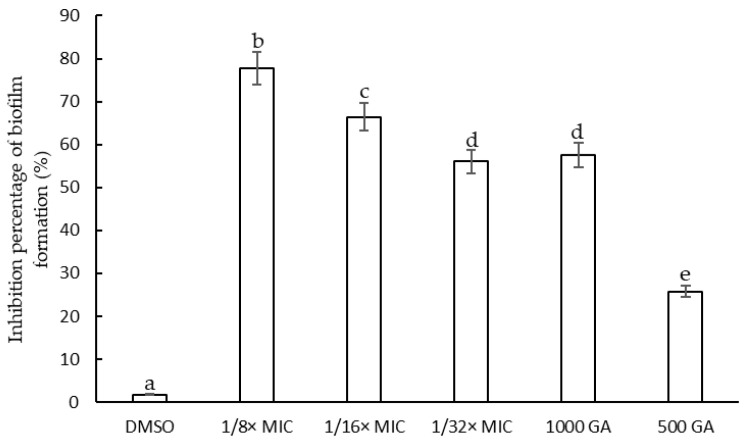
Percentage of inhibition of biofilm formation of *S. epidermidis* at different sub-MIC concentrations (1/8× MIC– 1/32× MIC) of *F. densa* extract and gallic acid (GA) (1000 and 500 µg/mL). Bars with different letters indicate significant differences (*p* < 0.05).

The extracts showed Inhibition of cell adhesion during biofilm formation. The effect of the extracts was evidenced by a reduction in biofilm biomass produced with respect to the control (Figure 4). The extract at a concentration of 1/8× MIC presented the highest percentage of inhibition of initial cell adhesion (77.75%), followed by that at concentration adjusted to 1/16× MIC (66.45%) and 1/32× MIC (56.01%). The highest concentration of GA presented a low inhibitory effect of biofilm formation similar to that of the lowest concentration of extract assayed (57.01%).

Table 2 shows the percentages of metabolic activity reduction with respect to the control during biofilm formation. The *F. densa* extract at 1/8× MIC and 1000 µg/mL of GA, used as reference compound, showed the highest percentage of inhibition of metabolic activity. In addition, the inhibition of metabolic activity decreased when the dilution increased. The concentration of 500 µg/mL of GA presented a reduction in metabolic activity as that of the medium whose concentration was 1/16× MIC (42.94%).

#### 2.3.2. Effect of *F. densa* Extract on Preformed Biofilm and Metabolic Activity

The effect of different concentrations of the *F. densa* extract on preformed biofilm was evaluated. After 24 h of incubation, the concentrations tested exhibited a low capacity to eradicate the preformed biofilm. The highest percentage of inhibition was shown by the medium with a concentration of 1/8× MIC (25.66%), while the highest dilutions tested and the medium supplemented with GA (1000 µg/mL) presented a removal capacity below 10%. Finally, the concentration of 500 µg/mL GA did not present activity to remove preformed biofilm. An effect of DMSO can also be observed in the removal of the preformed biofilm (12%) so that the solvent could favor the effect of the tested extract. On the other hand, Table 2 shows metabolic activity inhibition percentages obtained via MTT assay. The maximum reduction in metabolic activity was registered after the preformed biofilm was in the presence of 1/8× MIC (22.49%), which was similar to the reduction produced with 500 µg/mL of the reference compound GA (26.59%).

#### 2.3.3. Reduction in Extracellular Polymeric Substances (EPS) and Proteins during Biofilm Formation

The structure of the biofilm is formed by a matrix composed of extracellular polymeric substances, such as polysaccharides, proteins, and extracellular DNA [30]. Additionally, this external matrix, called slime, confers stability and resilience to the biofilm [31]. The cells are embedded in this matrix, which serves as a protective barrier against the attack of antimicrobial compounds or against the host’s own defense mechanisms. A mechanism through which the EPS content is reduced would allow the biofilm to be destabilized and favor antimicrobial action [32].

*F. densa* extracts caused a reduction in the EPS formed by *S. epidermidis*. The concentration of 1/8× MIC of extracts was able to reduce 33.0% of the EPS formed with final EPS concentrations of 27.32 mg/mL. This effect was similar to that observed with 1000 µg/mL GA (final concentration of 26.64 mg/mL). On the other hand, 1/16× MIC and 500 µg/mL of GA did not have a different effect between them, producing a reduction in EPS between 17.0 (33.7 mg/mL) and 19.0% (33.26 mg/mL), respectively. In extract-free or GA-free conditions, *S. epidermidis* produced an EPS concentration of 40.78 mg/mL. Neither DMSO nor 1/32× MIC concentration exhibited reduction in EPS formed relative to the control.

One of the important constituents of EPS are proteins. The reduction in protein content was quantified via the Bradford method. At the highest tested concentration (1/8× MIC), the reduction in protein content was 61.91% (8.25 µg/mL). This effect was greater than that observed for GA at a concentration of 1000 µg/mL, which produced a reduction in 40.98% (12.78 µg/mL), and 500 µg/mL of GA barely reduced the protein content by 16.74%. The solvent did not present significant differences with respect to the control and, under these conditions, *S. epidermidis* produced a concentration of 22.0 µg/mL of proteins.

#### 2.3.4. Microscopic Observation of Biofilm Formation

**Figure 5:**
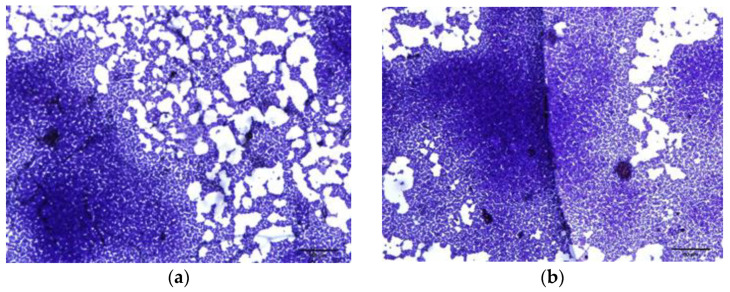

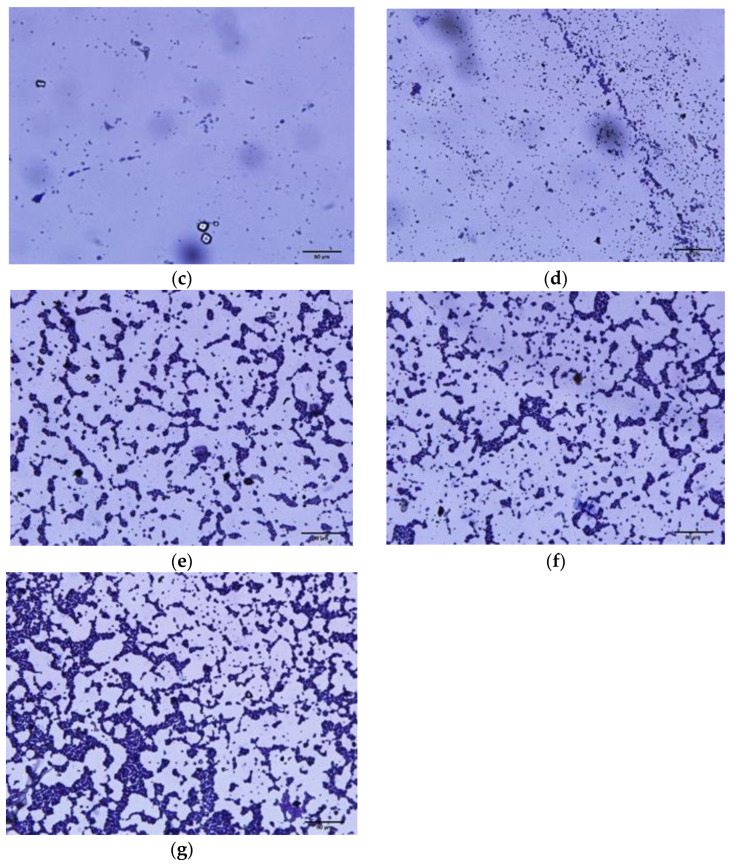
Micrographs obtained via light microscopy showing the reduction in biofilm adherence of *S. epidermidis* on glass slides at different concentrations of *F. densa* extract. (**a**) control (without extract), (**b**) solvent control (DMSO), (**c**) 1/8× MIC, (**d**) 1/16× MIC, and (**e**) 1/32× MIC of *F. densa* extract, (**f**) 1000 µg/mL and (**g**) 500 µg/mL of GA.

The biofilm inhibitory activity of the extracts of *F. densa* and GA was confirmed via visualization using light optical microscopy. A reduction in the coverage of the biofilm formed on the glass surface was clearly observed in those slides that were incubated in the presence of the extract and gallic acid, while in the control medium and in the medium with 1/32× MIC, it was observed that the surface was entirely covered with cellular aggregates (Figure 5).

## 3. Discussion

### 3.1. Extraction Methods and Chemical Composition of Fabiana Species

Conventional extraction techniques such as maceration or infusions have been widely used for the extraction of compounds of plants and their standardization. However, these methods usually have some disadvantages. They require a longer extraction time and a larger amount of plant tissue; in addition, organic solvents are used, which could have a negative effect on human health and the environment, and, in some cases, heating is necessary, which produces an extra cost of energy [33]. For this reason, strategies such as extraction with unconventional methods have been developed to overcome these disadvantages [34]. UAE is characterized by greater extraction efficiency and, since it does not require much time, it can also be employed at ambient pressures; in addition, this procedure is cheaper than traditional methods [35]. However, the cost of the equipment is quite high compared to the cost of the equipment needed to carry out macerations.

To date, a limited number of studies on the antibacterial effectiveness of the *Fabiana* species described in this work have been carried out. The collection of extracts of *Fabiana* species from the ecoregion of the Monte de Sierras y Bolsones (2200–3000 m above sea level) obtained via UAE was shown for the first time in this article. Some authors [23,24] reported that alcoholic extracts of *F. densa*, *F. patagonica* and *F. punensis*, collected from the Puna region of Argentina, located between 3700 and 4800 m.a.s.l., obtained via M, showed higher concentration of TPC compared with the species of this work. The difference in the concentration of the phenolic compounds could be due to the difference in altitudes from where the samples were collected. Some authors reported an increase in the content of secondary metabolites in plants associated with growth at higher altitudes. These increases could be related to environmental abiotic factors such as low temperatures and UV-B radiation, which increase at higher altitudes [36,37,38].

The UAE is an economical and eco-friendly method that allows one to obtain phenolic compounds in less extraction time compared to other conventional methods such as maceration. For example, using the response surface model, the conditions were optimized to obtain *Eucalyptus marginata* L. extracts with the highest TPC content, in which the optimal extraction time for UAE was shorter (49.9 min) than that required for maceration (88 min) [39]. In the same way, the optimization of the extraction of TPC from ethanolic extracts (60% *v*/*v*) of *Robiniae pseudoacaciae* flowers via the maceration, Soxhlet and UAE methods presented optimal extraction time as being higher for maceration (24 h), followed by that required for Soxhlet (6 h) and finally that for UAE (0.5 h) [40]. Many authors agree that UAE improves the extraction rate, that is, the extraction time is shorter compared to other conventional methods [41,42,43]. Our results are in agreement with what was published by these researchers. The UAE allowed one to obtain extracts with higher yield, since, although the TPC concentration is similar to that of the extracts obtained via M, the extraction time in UAE is significantly less (30 min) compared to the extraction time required with M (7 days). Furthermore, no differences were observed in the compounds extracted in both methods, evidenced by the HPLC profiles. In other words, this unconventional method allows the extraction of the same amount and composition in a shorter extraction time.

### 3.2. Antimicrobial and Antibiofilm Activity of F. densa against S. epidermidis

In this work, the antimicrobial and antibiofilm activity of the ethanolic extract of *F. densa* against resistant *S. epidermidis* clinical isolate was demonstrated.

It is widely accepted that the MIC corresponds to the lowest concentration of the antimicrobial that produces the inhibition of the microorganism and is one of the ways of expressing the antimicrobial activity of a compound [44]. It is also a criterion for selecting new antimicrobials. Crude extracts that present a MIC below 100 µg/mL [45], and less than or equal to 10 µg/mL for isolated compounds from extracts [46], are generally accepted as antimicrobials with promising properties. The *F. densa* extract presented an MIC of 75 µg/mL, suggesting that it has appropriate and promising antimicrobial activity. To date, little has been reported on the antimicrobial activity of this plant against this opportunistic pathogen. Nevertheless, researchers isolated and identified two diterpenes derivates, oxalyl and succinyl ester, from acetone extracts of *F. densa* variety *ramulosa* with antimicrobial activity against *S. epidermidis* ATCC 12228. The authors revealed that these compounds inhibited the growth of the population of *S. epidermidis* by 50 and 80%, respectively, at the highest concentration assayed (64 µM) [26]. There are various mechanisms through which agents can combat microbial infections, primarily by inducing bacterial cell death and targeting different areas, such as specific inhibition of cell wall synthesis, destabilization of membrane components, inhibition of DNA replication, or restriction of protein synthesis [47]. An increase in absorbance measurements at 260 nm and the release of proteins serve as indicators of irreversible damage to membrane integrity, implying a loss of internal cellular materials such as nucleic acids, cytoplasmic material, and essential proteins necessary for cell growth [48]. As it was demonstrated in the results section, the presence of the *F. densa* extracts produced an increase in these indicators in the external environment, which would mean that the extracts of *F. densa* potentially possess an antimicrobial mechanism that would act by damaging the integrity of the membrane.

*S. epidermidis* is a pathogen able to produce biofilm. This property favors resistance to antibiotics, which makes it difficult to treat infections caused by this microorganism. Studying alternatives for inhibiting biofilm formation is an interesting prospect for the treatment of these infections associated with this virulence factor. *F. densa* extract showed a high percentage of inhibition of *S. epidermidis* biofilm formation at sub-MIC concentration. Some authors reported that aqueous extracts from different parts of plants present in semi-arid areas of Brazil (Caatinga) were screened against the formation of biofilm produced by the *S. epidermidis* strain ATCC 35984. It was found that at a concentration of 0.4 mg/mL of the extracts, the percentages of inhibition were in a range between 56.7 and 67.3% without producing inhibition of microbial growth. At higher concentrations (4 mg/mL), these extracts inhibited the growth of the bacteria by at least 40% [49]. The anti-biofilm effect of ethanolic extracts from different Thai herbal recipes on *S. epidermidis* ATCC 35984 was evidenced through the MTT reduction assay. The authors found that a combination of *Maranta arundinacea* L. (Rhizome) *Oroxylum indicum* Vent. (Bark) *Commelina benghalensis* L. (whole plant) completely inhibited biofilm formation at a concentration of 250 µg/mL, while the the combination of *Curcuma longa* L. (Rhizome), *Areca catechu* L. (Seed) *Oryza sativa* L. (Seed), *Garcinia mangostana* L. (Pericarp) showed inhibition of activity between 30 and 40% for concentrations between 0.63 and 5 µg/mL without inhibiting the growth of the bacterial strain [50]. Additionally, it was found that hydroalcoholic extracts of hops from *Humulus lupulus* L. variety *cascade* presented high percentages of inhibition of biofilm formation between 95 and 99% against the strains of *S. epidermidis* SE009 and *S. epidermidis* 317 at a concentration of 1/2× MIC; even at a concentration of 1/4× MIC, the hop extract showed an inhibition of 65% [51]. The *F. densa* extracts obtained via UAE presented an effect similar to those reported by these researchers, exhibiting a high percentage of inhibition of biofilm biomass formation, close to 80%, and a reduction in metabolic activity, determined via the assay of MTT, greater than 60%.

GA has been shown to exhibit antimicrobial activity against bacterial pathogens with MIC values ranging from 250 to 1000 µg/mL. Specifically, against the *S. epidermidis* 12228 strain, the MIC value was >1000 µg/mL [52]. In this study, it was also detected that at concentrations below 1000 µg/mL, there was no inhibition of microbial growth with respect to the control. Additionally, GA has been reported as an inhibitor of biofilm formation against other Gram-positive bacterial genera such as *Staphylococcus aureus* [53], *Streptococcus mutans* [54], and Gram-negative bacteria such as *Escherichia coli* [55] and *Pseudomona aeruginosa* [56]. However, in this research, the inhibitory activity of biofilm formation by GA against *S. epidermidis* was reported for the first time.

The ability of some agents to eradicate preformed biofilm is an interesting prospect for the treatment of advanced infections. The *F. densa* extract presented a high percentage of inhibition of biofilm formation; however, the percentage of eradication of the preformed biofilm was low. Similar results were observed with a soybean extract against the *S. epidermidis* ATCC 35984 strain, which exhibited a high percentage of inhibition of biofilm formation (77%), while it did not show the ability to eradicate a biofilm formed after treatment (24 h) [57]. This suggests that the activity of the *F. densa* extract acts mainly during the early stages of biofilm formation. Some authors evaluated the effect of the extract on the eradication of preformed biofilm using MTT assay. They found that after 24 h of incubation with ethanolic extract of Thai herbals recipes at a concentration of 500 µg/mL and 20 µg/mL, it presented a reduction of more than 90% of the 7-day-old biofilms produced by *S. epidermidis* ATCC 35984 [50]. The ability of alpha mangostin to eradicate the performed biofilm produced by the *S. epidermidis* strain ATCC 35984 was also reported. The reduction in the biomass of the mature biofilm was 38 to 47% at high concentrations of the compound, 8× MIC and 16× MIC, respectively. The authors propose that the reduction in the preformed biofilm is related to the high concentrations of the extract tested, which presents bactericidal activity. However, this activity of killing bacteria is lower compared to biofilms with a longer maturation time (24 h). The low concentrations tested could explain (sub-MIC) the low capacity of the *F. densa* extract to reduce the preformed biofilm [58].

On the other hand, the *F. densa* extract was able to produce an important reduction in EPS and proteins content in a dose-dependent manner during the formation of the biofilm. These results are in accordance with some reports, i.e., umbelliferone (500 µg/mL) produces an inhibition of 83% of the biofilm biomass of methicillin-resistant strain *S. epidermidis* ATCC 35984 and a reduction in carbohydrate and protein content of 57 and 27%, respectively [59]. Sessile cell adherence to surfaces favors the resistance of these bacteria, which prevents or reduces this adherence, thus becoming a challenge for medicine. Optical microscopy made it possible to visualize and validate the reduction in adhesion produced by the dose-dependent extract of *F. densa* and GA on the biofilm formed by *S. epidermidis* on glass.

## 4. Materials and Methods

### 4.1. Plant Material

Aerial part of *F. densa*, *F. patagónica* and *F. punensis* were collected in February 2021 from Monte de las Sierras y Bolsones region in the Argentinean Northwest area (26°36′42″ S 65°50′26″ W). The identification of plant materials was carried out by the botanist Ana Soledad Cuello. Both the Fundación Miguel Lillo Herbarium (LIL) and INBIOFIV received the voucher specimens.

### 4.2. Plants Extracts Preparation

The preparation of the extracts was carried out using two methods: maceration (M) and ultrasound-assisted extraction (UAE). The plant material was previously dried and ground. For comparative purposes, ethanol 80° was used as an extraction solvent, in addition to a liquid–solid ratio of 20 (5 g of dry plant tissue in 100 mL of solvent), for both extraction methods.

Maceration was carried out for 7 days of incubation with shaking at 40 cycles per minute at 40 °C. The UAE was performed using the ultrasonic homogenizer (UP200St, 200 w, 26 KHz HielscherUltrasonics GmbH, Teltow, Germany). Extraction was carried out in an ice bath. The available bibliography was used to set the extraction variables. Amplitude: 70% [60]; extraction time: 30 min [61]; and constant pulse mode (5 s on/ 5 s off) [62].

The extracts were then concentrated using a rotary evaporator at 40 °C after being filtered with Whatman No. 1 filter paper. The extracts were then dissolved in dimethyl sulfoxide (DMSO) and stored at −20 °C.

### 4.3. Phytochemical Screening

#### 4.3.1. Phenolic Compound Determination

The Folin–Ciocalteu method was used to calculate the total phenolic compound (TPC) content [63]. The results were presented as equivalents of gallic acid. (GAE). A spectrophotometric approach was used to quantify the content of total flavonoids (TF). Quercetin equivalents (QE) were used to express the results [64].

#### 4.3.2. HPLC Analysis

A HPLC fingerprint was performed in order to compare the profile of compounds from *Fabiana* extracts obtained via maceration vs. UAE. The analysis was carried out according to with some modification. Briefly, an HPLC system consisting of a Waters 1525 Binary HPLC Pumps system with a 1500 Series Column Heater, a manual injection valve with a 20 µL loop (Rheodyne Inc., Cotati, CA, USA) and a Waters 2998 photodiode array detector (PDA) were used to analyze the extracts (220–540 nm). Samples (2 mg/mL) were run through a XBridgeTM C18 column (4.6 mm × 150 mm, 5 µm; Waters corporation, Milford, MA). A gradient system was used as mobile phase: 0.1% acetic acid in water (A) and 0.1% acetic acid in methanol (B). The gradient system was performed with a flow rate of 0.5 mL/min as follows: A 90% + B 10% from 0 to 35 min, A 43% + B 57% from 35 to 45 min, and B 100% from 45 to 65 min [65]. The fingerprints obtained at 254 nm of extracts of each plant species were compared.

### 4.4. Bacterial Strain

*S. epidermidis* (INBIOFIV S11) clinical isolates of human patients from Hospital Dr. Nicolás Avellaneda, San Miguel de Tucumán, Tucumán, Argentina, were used. The identification of the strain was carried out using biochemical profiles according to the recommendations of the Manual of Clinical Microbiology [66].

The organism was maintained in a Müeller–Hinton (MH) medium supplemented with 30% (*v*/*v*) glycerol at −20 °C. Before testing, the suspensions were transferred to trypticase soy agar and incubated aerobically overnight at 37 °C.

### 4.5. Antimicrobial Susceptibility Testing

Disk diffusion method with antibiotics was used to determine in vitro antimicrobial susceptibility testing in accordance with the guidelines recommended by the Clinical and Laboratory Standards Institute (CLSI). The assayed antibiotics were penicillin (10 μg), oxacillin (1 µg) erythromycin (15 μg), clindamycin (2 μg), tetracycline (30 μg), gentamicin (10 μg), chloramphenicol (30 μg) ampicillin (2 μg) and rifampicin (5 μg). *S. aueus* ATCC 29213 was used as control strain in accordance with CLSI breakpoints. The minimum inhibitory concentration value of vancomycin was determined with the standard agar dilution method recommended by the CLSI [67].

### 4.6. Minimal Inhibitory Concentration (MIC)

MIC values of *Fabiana* extracts were determined using the serial agar macrodilution method. Briefly, 500 µL of serial two-fold dilution of each stock extract solution was added to 4.5 mL of MH medium to obtain a final concentration range of 1200– 7.5 µg GAE/mL. Subsequently, the culture media were inoculated with 2 µL spots of a bacterial suspension previously adjusted to a concentration of 10^5^ CFU/mL (OD_560nm_: 0.08 diluted 1:100). The plates were then incubated at 37 °C for 20 h. Control of DMSO was also carried out. Culture without extract and without DMSO was considered as negative control. MIC was defined as the lowest concentration of extract at which no colony was observed after incubation.

### 4.7. Viability Cell Analysis and Release of Cellular Constituents

The growth curve assay was performed to determine the effect of the different concentrations of *F. densa* extract on the viability of *S. epidermidis*. Serial two-fold dilutions of stock extract solution were prepared in 2 mL of trypticase soy broth (TSB) to obtain a final concentration range of 16× MIC–1/32× MIC µg/mL of GAE and inoculated with the bacterial suspension to reach a final concentration of 1.5 × 10^5^ CFU/mL. Cultures were incubated at 37 °C for 24 h. Aliquots were taken at 4, 8, 12 and 24 h diluted and seeded on MH agar in order to determine the viable cell concentration expressed as CFU/mL. TSB medium without extract was used as positive control. The effect of higher DMSO concentrations also was evaluated.

### 4.8. Damage to Membrane Integrity

Membrane integrity was examined by determining the release of cellular constituents in those extracts whose concentration was higher than the MIC (MIC-16x MIC). For this, the cultures were centrifuged at 9000× *g* for 10 min every 4 h. Supernatants were collected, and absorbance was measured at 260 nm [68]. The content of released protein was determined using the Bradford method [69]. Blanks were used for each of the conditions to eliminate the interference produced by the presence of the extract in the culture medium. TSB without extract was used as the negative control of the reaction.

### 4.9. Determination of the MIC of Biofilm Formation

The aim of this assay was to determine the minimum concentration at which initial bacterial cell attachment is inhibited. For this purpose, a bacterial suspension was prepared and the absorbance was adjusted to 0.08 at 560 nm and diluted 1:100 in 200 µL of TSB supplemented with 1% glucose to reach a final concentration of 1.5 × 10^6^ CFU/mL. The assay was carried out in TSB supplemented with sub-inhibitory concentrations (sub-MIC) of *F. densa* extract (1/2 MIC–1/32 MIC). As a positive control, TSB without extract was used. The effect of the highest concentration of DMSO tested was also considered. Gallic acid (GA), a phenolic acid present in several vegetable species, is reported as a natural inhibitor of biofilm formation [70]. Therefore, in this trial, the inhibition capacity of the *F. densa* extract was compared with different sub-MIC concentrations of GA (500, 1000, 2000 µg/mL). The assay was carried out in 96-well microplates. Microcultures were incubated at 37 °C for 24 h. Subsequently, the planktonic cells were carefully aspirated to remove them from the wells and washed three times with phosphate-buffered saline (PBS-pH 7.4). The microplate was left to air-dry for 24 h; subsequently, the wells containing the biofilm formed were stained with 200 µL of a 0.3% (*w*/*v*) crystal violet solution for 15 min at room temperature. The wells were washed three times with PBS to remove unabsorbed dye. Subsequently, glacial acetic acid (30% *v*/*v*) was used to dissolve crystal violet and the titer plate was read at 570 nm. The percentage of biofilm inhibition was calculated using Equation (1) [71]:

Biofilm inhibition (%) = [(Control OD − Treated OD)/Control OD] × 100(1)

### 4.10. Determination of Preformed Biofilm Eradication Capacity

In order to evaluate the eradication capacity of the biofilm preformed (maturation phase), an incubation of 48 h was carried out. Briefly, microplates containing 200 µL of TSB supplemented with 1% glucose inoculated with a bacterial suspension at a final concentration of 10^6^ CFU/mL were incubated for 24 h at 37 °C to promote adherence and biofilm formation. Subsequently, the exhausted medium and the planktonic cells were discarded, and the adhered biofilm was rinsed with PBS three times to eliminate the non-adhered cells. Each well was reloaded with TSB supplemented with 1% glucose spiked with sub-MIC concentrations of *F. densa* extract (1/2x MIC–1/32x MIC). TSB supplemented with 1% glucose without inoculation was considered a negative control. The highest concentration of DMSO tested and the different concentrations of gallic acid mentioned in the previous section were also considered. After 24 h of incubation, the medium was discarded and washed three times with PBS to remove non-adherent cells. Subsequently, the formed biofilm was stained according to the crystal violet staining protocol described in the above section. The test was performed in quintuplicate, and the percentage of biofilm removal was calculated with Equation (1) [72].

### 4.11. Measurement of the Biofilm Metabolic Activity of the Biofilm

Respiratory activity both in the initial phase and in the maturation phase of biofilm was measured using the 3-[4, 5- dimethylthiazol-2-yl]-2, 5- diphenyltetrazolium bromide (MTT) reduction assay. After 24 (attachment inhibition) and 48 h (removal of the biofilm formed) of incubation, planktonic or loosely attached cells were carefully aspirated to be discarded and the wells were washed with PBS three times. Then, 100 µL of MTT solution in PBS (5 mg/mL) was added to each well and incubated at 37 °C for 4 h. Subsequently, the insoluble purple precipitate produced by dehydrogenase enzymes present in living cells that hydrolyze MTT was further dissolved via the addition of 100 µL of DMSO. Absorbance measurements were obtained at 570 nm in a microplate reader. Each assay was performed in triplicate. TSB medium without extract was considered a positive control [73].

### 4.12. Extraction and Quantification of Extracellular Polymeric Substances (EPS)

To obtain the formed EPS, 1 mL of *S. epidermidis* culture (final concentration: 10^6^ CFU/mL) was incubated in TSB supplemented with 1% glucose in the presence and absence of the different sub-MIC concentrations mentioned above for 24 h at 37 °C. After incubation, the medium with planktonic cells was separated and centrifuged at 9000× *g* for 10 min to separate the cells from the medium and thus obtain a cell-free supernatant, which contains free EPS. On the other hand, the wells containing the biofilm formed were rinsed with PBS three times to eliminate planktonic cells and weakly adhered cells. Then, 500 µL of TE buffer (10 mM EDTA in 10 mM Tris buffer) was added and incubated for 24 h at 4 °C. Subsequently, the content of each well was taken and centrifuged at 9000× *g* for 10 min to eliminate the cells. After that, three times the volume of cold acetone was added to both the cell-free supernatant (free EPS) and the supernatant obtained from the wells (cell-bound EPS), and incubated for 24 h at 4 °C. The samples were centrifuged at 9000× *g* for 10 min and the supernatant was separated. The solution was incubated at room temperature to dry until evaporation of the acetone [32]. Subsequently, the precipitates were resuspended in TE buffer to quantify the total EPS content using the phenol sulfur method (Nielsen 2010). Quantification was performed using a glucose 1 mM solution to perform a calibration curve. Absorbances were measured at 490 nm. Finally, the protein content was quantified according to Bradford method [69]. A bovine serum albumin (BSA) solution of 0.5 mg/mL was used as a standard solution to perform a calibration curve.

### 4.13. Biofilm Inhibition Optical Microscopy Visualization

The reduction in biofilm formation was validated using light microscopy. For this purpose, the protocol described in Section 4.9 with some modifications was used. Briefly, 1 mL of TSB supplemented with glucose 1% previously inoculated with bacteria (final concentration 10^6^ CFU/mL) was placed in the wells of 24-well microplates with and without the presence of the *F. densa* extract at different sub-MIC concentrations. A clean and previously sterilized 1 cm × 1 cm glass coverslip was placed in each well. After 24 h of incubation at 37 °C, the depleted medium with planktonic or poorly attached cells was aspirated, the coverslips were washed with PBS three times and allowed to dry in the air. Subsequently, they were stained with crystal violet solution (0.3% *w*/*v*) for 15 min. Cover slips were washed with sterile distilled water to remove excess staining and then air-dried. Stained glass pieces were placed on slides with the biofilm on top of the glass slide. Biofilms were evaluated and confirmed using light microscopy at 100× magnification [74].

### 4.14. Statical Analysis

All the experiments were carried out in triplicate except for the biofilm quantification assays using the crystal violet method, which were carried out in quintuplicate. Experiments were performed with at least two technical replications. The results were presented as the mean ± standard deviation (SD). Statistical significance was determined using the one-way ANOVA method followed by Tukey’s post hoc test using the statistical software InfoStat version 2017.1.2. Values were considered significantly different at *p*-value < 0.05.

## 5. Conclusions

The UAE is an extraction method of TPC from *Fabiana* species that is more efficient than M in ethanol since it entails a shorter extraction (30 min) compared to that when employing M (7 days). The MIC values for both extraction methods did not present differences for the same species. However, the *F. densa* extract showed the lowest MIC value against *S. epidermidis* of the three species tested. The presence of the extracts produced an increase in the release of cellular constituents such as DNA and proteins; therefore, the extract could exert an antimicrobial mechanism of action involved in the rupture of the cell membrane. On the other hand, concentrations below 1/8x MIC did not affect cell growth and showed inhibitory activity on biofilm formation and reduction in cell metabolic activity in a dose-dependent manner. However, the extracts showed a low reduction in the preformed biofilm. The presence of the extracts reduces the production of EPS and proteins significantly during the biofilm formation phase. Finally, extracts reduce adhesion to inert surfaces, both hydrophilic (glass) as hydrophobic (polystyrene microplates); thus, they could potentially be used for the treatment of infections associated with medical devices. This study supports the potential use of *F. densa* extracts for the treatment of infections caused by *S. epidermidis* through antimicrobial therapy or as anti-virulent agent inhibitors of biofilm formation, as there is a pressing need to find alternatives for the treatment of infections caused by bacteria resistant to antibiotics. At our laboratory, more investigation is being carried out to clarify the potential mechanisms preventing the production of biofilms.

## Figures and Tables

**Table 1:** Yield of dry extract, total phenolics compounds and total flavonoids of Fabiana species extracts obtained via maceration and UAE. Values are presented as mean ± standard deviation (*n* = 3).

Plant	Total Phenolics Compounds (µg GAE^1^/mL)		Total Flavonoids (µg QE^2^/mL)		Yield of Dry Extract (mg/mL)	
	Maceration	UAE	Maceration	UAE	Maceration	UAE
*F. densa*	1615.41 ± 61.42 ^aA^	1586.03± 86.47 ^aA^	220.53 ± 6.76 ^aA^	143.59 ± 10.88 ^aB^	16.80 ± 0.43 ^aA^	13.43 ± 0.05 ^aB^
*F. patagonica*	2101.11 ± 28.82 ^bA^	1824.17 ± 33.26 ^bB^	165.06 ± 3.53 ^bA^	86.24 ± 5.29 ^bB^	15.20 ± 0.62 ^bA^	8.43 ± 0.05 ^bB^
*F. punensis*	1930.38 ± 22.17 ^cA^	1872.06 ± 44.35 ^bA^	234.76 ± 5.00 ^cA^	180.35 ± 2.94 ^cB^	12.86 ± 0.60 ^cA^	10.53 ± 0.4 ^cB^

Different lowercase letters indicate significant differences for the same column. Different capital letters indicate significant differences between two columns, that is, for the two extraction methods for the same extract. (*p* < 0.05). GAE^1^: gallic acid equivalents QE^2^: quercetin equivalents.

**Table 2:** Percentage of inhibition of metabolic activity during biofilm formation and on preformed biofilm. Data are presented as means ± standard deviation (*n* = 3).

Treatment	Biofilm Formation Inhibition (%)	Biofilm Preformed Eradication (%)
DMSO	5.89 ± 2.57 ^a^	0.24 ± 0.44 ^a^
1/8× MIC	66.56 ± 0.43 ^b^	22.49 ± 7.14 ^b^
1/16× MIC	42.53 ± 2.26 ^c^	19.38 ± 7.15 ^b^
1/32× MIC	30.33 ± 5.18 ^d^	10.15 ± 3.51 ^c^
1000 GA	67.04 ± 3.60 ^b^	40.46 ± 4.50 ^d^
500 GA	42.95 ± 1.27 ^c^	26.59 ± 2.55 ^b^

Different letters for the same column denote statistically different differences (*p* < 0.05).

## Data Availability

Data are available from the corresponding author upon reasonable request.
